# Lipopolysaccharide-coated CuS nanoparticles promoted anti-cancer and anti-metastatic effect by immuno-photothermal therapy

**DOI:** 10.18632/oncotarget.22331

**Published:** 2017-11-06

**Authors:** Bian Jang, Li Xu, Madhappan S. Moorthy, Wei Zhang, Ling Zeng, Mingyeong Kang, Minseok Kwak, Junghwan Oh, Jun-O Jin

**Affiliations:** ^1^ Shanghai Public Health Clinical Center, Shanghai Medical College, Fudan University, Shanghai, China; ^2^ Marine-Integrated Bionics Research Center, Pukyong National University, Busan, South Korea; ^3^ Department of Biomedical Engineering and Center for Marine-Integrated Biomedical Technology (BK21 Plus), Pukyong National University, Busan, South Korea; ^4^ Interdisciplinary Program of Biomedical Mechanical & Electrical Engineering, Pukyong National University, Busan, South Korea; ^5^ Department of Chemistry, Pukyong National University, Busan, South Korea

**Keywords:** lipopolysaccharide, copper sulfide nanoparticles, photothermal therapy, immunotherapy, anti-tumor

## Abstract

To meet the ultimate goal of cancer therapy, which is treating not only the primary tumor but also preventing metastatic cancer, the concept of combining immunotherapy with photothermal therapy (PTT) is gaining great interest. Here, we studied the new material, lipopolysaccharide (LPS) coated copper sulfide nanoparticles (LPS-CuS), for the immuno-photothermal therapy. We evaluated the effect of LPS-CuS for induction of apoptosis of CT26 cells and activation of dendritic cells. Moreover, the LPS-CuS and laser irradiation was examined anti-metastasis effect by liver metastasis model mouse *in vivo*. Through PTT, LPS-CuS induced elimination of CT26 tumor in BALB/c mice, which produced cancer antigens. In addition, released LPS and cancer antigen by PTT promoted dendritic cell activation in tumor draining lymph node (drLN), and consequently, enhanced the tumor antigen-specific immune responses. Finally, the primary tumor cured mice by LPS-CuS-mediated PTT completely resisted secondary tumor injection in the spleen and also prevented liver metastasis. Our results demonstrated the potential usage of LPS-CuS for the immuno-photothermal therapy against various types of cancer by showing the clear elimination of primary colon carcinoma with complete prevention of spleen and liver metastasis.

## INTRODUCTION

To achieve success in cancer therapy, great endeavors have been devoted to cancer research all around the world. Notwithstanding these efforts, metastasis is the main obstacle for the complete treatment of cancer, and it causes majority of death due to cancer because metastatic cancer is hard to diagnosis and treat [1]. In recent years, the interest in immunotherapy is greatly increasing due to the possibility that the patient's own immune system can notice, target, and attack tumor cells in both the primary and metastatic sites [2]. Various types of immunotherapy have been developed for and applied in cancer therapy including monoclonal antibody immunotherapy [3], adoptive cell transfer [4], checkpoint-blockade therapy [5], and cancer vaccines [6, 7]. Despite the infinite potential of immunotherapy, there still are some considerable limitations: high treatment cost [8], unpredictable variation in clinical treatment response of each individual [9], and some side effects, such as inflammation [10]. Therefore, a new type of immunotherapy that can be applied to various different conditions with high specificity is needed.

Photothermal therapy (PTT) has been developed as an alternative to or a supplement of conventional cancer therapy with the principle of ablation of cancer cells using the heat generated from photo-absorbing nanoparticles that absorb and convert a near-infrared (NIR) light energy into thermal energy [11, 12]. According to previous research, PTT with well-designed nanoparticles (such as gold nanoparticles, magnetic iron oxide nanoparticles, or carbon nanotubes) can effectively eliminate cancer cells through apoptosis, the process of programmed cell death [13–15]. PTT-induced activation of apoptosis signaling pathway of cancer cells such as caspase 8, 9, and 3 promotes the production of apoptotic bodies [13, 16], which can be phagocytosed by antigen-presenting cells (APCs) [i.e. dendritic cells (DCs) and macrophages] for antigen-specific immune activation [17–19]. However, without immunostimulatory molecules, DCs are not able to fully activate to induce antigen-specific cytotoxic T lymphocyte (CTL) activation [17, 20–22]. Therefore, the combination of immunostimulatory molecules and PTT will be the advanced method for cancer therapy, especially for the prevention of cancer metastasis through antigen-specific immune activation.

Lipopolysaccharide (LPS) is a well investigated immunostimulatory molecule [23–25]. LPS induces activation of DCs including CD8α^+^ and CD8α^−^ DCs in the mouse *in vivo* [25, 26]. Moreover, LPS promotes cross and direct presentation of antigens by CD8α^+^ and CD8α^−^ DCs, respectively, which consequently induces CTL and helper T (Th) cell activation [22, 25, 27]. In addition, LPS is heat stable [28]. Therefore, we hypothesized that LPS-coated copper sulfide nanoparticles (LPS-CuS) will be able to induce apoptosis of tumor cells through the CuS-mediated PTT, and free form of LPS together with apoptotic bodies will promote antigen-specific immune activation. We aimed to prove the effect of LPS-CuS in the immuno-photothermal therapy for the treatment of the primary tumor and prevention of its metastasis.

## RESULTS

### Preparation and characterization of LPS-CuS

**Figure 1:**
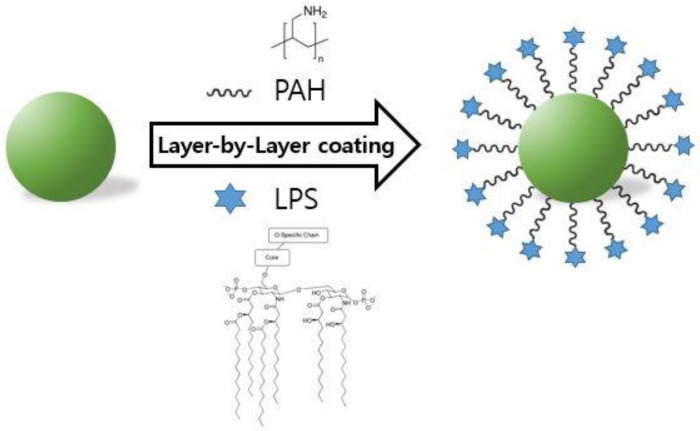
Schematic diagrams Structural illustration of LPS-CuS.

**Figure 2:**
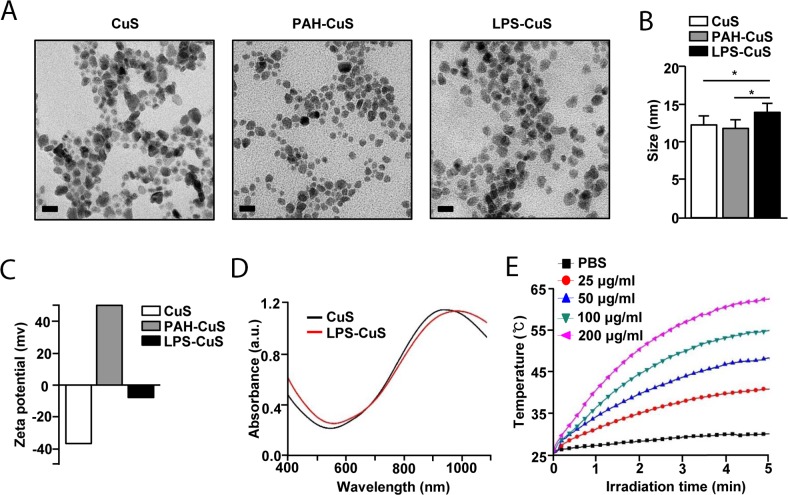
Characterization of F-CuS **(A)** FE-TEM images of CuS, PAH-CuS, and LPS-CuS. (Scale bars: 20 nm). **(B)** TEM corresponding size distribution of each coated nanoparticles. **(C)** Zeta potential values of each NPs. **(D)** UV-vis absorption of CuS (black) and LPS-CuS (red). **(E)** Photothermal heating curves of different concentrations of LPS-CuS dissolved in water, irradiated for 5 min with an 808-nm laser at a power density of 1 W/cm^2^.

To promote immuno-photothermal therapy against cancer, sodium citrate-stabilized CuS were synthesized and these were further coated with the poly-cationic and anionic substances [i.e., poly (allylamine hydrochloride) (PAH) and LPS] using the layer-by-layer (LbL) technique (Figure 1). Due to the possession of positively charged functional group, PAH was coated onto CuS as an intermediate linker for the additional coating of a negatively charged material, which also improves the stability of CuS [29, 30]. Based on this, coating of LPS on the outside of PAH-CuS was successfully performed via a strong electrostatic interaction between PAH (NH_3_^+^) and LPS (PO_3_^−^). According to the FE-TEM images, the size of spherical LPS-CuS (13.5 nm ± 1.7 nm) was slightly bigger than spherical CuS and PAH-CuS with an average size of 12 nm ± 1.1 nm (Figure 2A and 2B). Additionally, the crystallization and purity of synthesized CuS were confirmed by XRD and ED pattern results, which were compared with the standard data provided by the Joint Committee on Powder Diffraction Standards card (06-0464). While the strong and sharp peaks showed the fair crystallinity of the prepared CuS, the absence of other peaks proves that the sample is highly pure (Supplementary Figure 1A). To confirm the successful LbL coatings of PAH and LPS onto CuS, zeta potential was performed, and it was observed that negatively charged CuS (−39.12 mV) converted to a positive charge after PAH coating (+46.47 mV), which became negative again after LPS coating (−8.23 mV) (Figure 2C). To further analyze the coating of LPS and PAH onto CuS, the NPs were analyzed by FT-IR spectra (Supplementary Figure 1B). The peak of Cu-S stretching at 614 cm^−1^ appeared in CuS, PAH-CuS, and LPS-CuS spectra, indicating the formation of CuS. Due to the citrate, a stabilizer of CuS, C–O (1110 cm^−1^) and O–H (1389 cm^−1^) peaks were present in the CuS spectra. After coating the PAH onto the CuS, a new peak indicating the bending mode of amine (N–H) appeared at 1498 cm^−1^. After applying the LPS coating onto PAH-CuS, the broad peaks of C–O–C and C–O in rings of polysaccharide re-appeared at 1070 cm^−1^. The new peaks of asymmetric stretching P=O in phospholipids and amide C=O presented at 1232 cm^−1^ and 1545 cm^−1^, respectively. The peaks at 2850 cm^−1^ and 2920 cm^−1^, corresponding to CH_2_ vibration, also appeared. Thus, these data indicated that LPS has successfully coated on PAH-CuS. Quantification of LPS coated onto the NPs was performed by measuring the UV absorption of fluorescein isothiocyanate (FITC)-labeled LPS-CuS. FTIC-labeled LPS (4 mg/mL) was coated onto PAH-CuS and the uncoated FITC-LPS was isolated through centrifugation. Then UV absorbance of the free FTIC-LPS in the collected supernatant was measured and the concentration of free FITC-LPS was calculated based on the calibration curves of FITC-LPS with different concentrations. It was estimated that about 1.5 mg/mL of FITC-LPS was coated onto PAH-CuS (Supplementary Figure 2).

### Photothermal property of LPS-CuS

Among various admirable features of CuS, the ability to absorb light in NIR region (700–1100 nm) and convert this light energy into heat through the vibrational energy of the d–d transition of Cu^2+^ is one of the most remarkable characteristics [31]. The coating of LPS onto CuS did not affect the UV absorbance nor interrupted the photothermal heating efficiency of CuS (Figures 2D and Supplementary Figure 3). Next, we evaluated photothermal property of LPS-CuS with different concentrations under laser irradiation (1 W/cm^2^) at 808 nm for 5 min. LPS-CuS showed great temperature increases in the dose-dependent manner compared to PBS: the temperature of 100 and 200 μg/mL samples reached up to 55°C and 62°C, respectively, within 5 min, while PBS sample showed temperature increase up to 30°C (Figure 2E). Furthermore, photostability of LPS-CuS, another significant feature of NPs, was proved by performing 5 cycles of laser ON/OFF experiment. According to the results of heating curves of LPS-CuS (100 μg/mL) during 5 cycles laser ON/OFF irradiation and its UV-absorption spectra before and after the laser irradiation with no distinct decrease in the temperature elevation graph and absorbance, excellent photostability of LPS-CuS was observed (Supplementary Figure 4A and 4B). Therefore, these data indicated that LPS-CuS has photostability and photothermal effect for cancer therapy. In addition, the stability of LPS-CuS were analyzed by measuring UV-vis absorption of each sample dissolved in cell media and PBS before and after 2 h (Supplementary Figure 4C and 4D).

### LPS-CuS induced PTT against CT26

**Figure 3:**
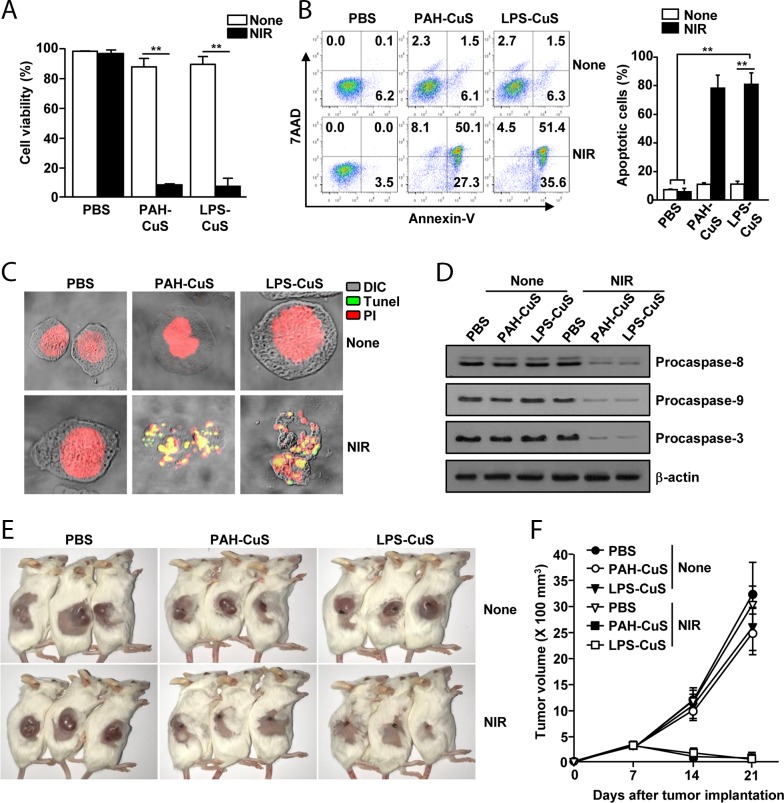
LPS-CuS with laser irradiation promoted anti-cancer effect against CT26 cells *in vitro* and *in vivo* CT26 cells were incubated with PBS, PAH-CuS, and LPS-CuS for 2 h, and the cells were treated with or without laser irradiation at 1 W/cm^2^ for 5 min and cultured for 24 h. **(A)** Cell viability of CT26 was measured by MTT assay; ^**^ p < 0.01. **(B)** Apoptosis were analyzed by annexin-V and 7AAD staining (left panel). Mean percentages of apoptotic cells were shown (right panel), ^**^ p < 0.01. **(C)** Nuclear degradation was analyzed by Tunel assay. **(D)** The expression levels of procaspase-8, -9, and -3 were measured by western blotting analysis. β-actin were used as a loading control. **(E-F)** BLAB/c mice were subcutaneously inoculated with 1 × 10^6^ CT26 cells. The mice were intratumorally injected with PBS, PAH-CuS, and LPS-CuS for 2 h 7 days after tumor injection and were treated with or without laser irradiation for 5 min. (E) The sizes of tumor mass on day 21 after tumor injection are shown. (F) CT26 tumor growth curves for the mice. Data are from the analyses of six individual mice (three mice per experiment, for a total of two independent experiments).

Because LPS-CuS showed great photothermal property, we next examined whether LPS-CuS can induce cancer cell death under laser irradiation. Before measuring anti-cancer effect of LPS-CuS in cancer cell, we examined cytotoxic effect of LPS-CuS in L132 cells, human embryonic pulmonary epithelial cells, and found that 100 and 200 μg/ml LPS-CuS did not induce cell death of L132 cells (Supplementary Figure 5A). This data indicated that LPS-CuS without laser irradiation is not toxic in the normal cells. We next examined LPS-CuS-mediated PTT effect in CT26, mouse colon carcinoma cells. The cells were treated with 25, 50, 100 and 200 μg/ml LPS-CuS for 2 h, and the cells were treated with laser irradiation (1 W/cm^2^, 808 nm laser) for 5 min. Without laser irradiation, the cell viability was not decrease, while 100 and 200 μg/ml LPS-CuS treatment with laser irradiation induced significant decreases in the cell viability (Supplementary Figure 5B). Next, CT26 were treated with PBS, 100 μg/ml PAH-CuS, and 100 μg/ml LPS-CuS for 2 h, and irradiated with a 808 nm laser (1 W/cm^2^) for 5 min. Laser irradiated PAH-CuS and LPS-CuS treatment substantially reduced cell viability, while the cell viability of PAH-CuS and LPS-CuS treatment without laser irradiation did not significantly change the cell viability 24 h after treatment (Figure 3A). Moreover, the laser irradiation in PAH-CuS and LPS-CuS-treated cells promoted significant increases in the apoptotic cell death (Figure 3B). In addition, the laser irradiation in PAH-CuS- and LPS-CuS-treated cells showed positive staining with Tunel reagent (Figure 3C), which indicates extensive DNA degradation and production of apoptotic bodies because late apoptosis displayed degradation of nuclear and developed apoptotic bodies [32]. For the further evaluation of PAH-CuS- and LPS-CuS-induced apoptosis of CT26 cells, we measured caspase signaling pathway after irradiating the laser in PAH-CuS and LPS-CuS-treated cells because degradation of pro-caspase proteins plays a central role in the execution of cell apoptosis [33, 34]. The treatment of PAH-CuS and LPS-CuS with laser irradiation induced marked decreases in the protein levels of procaspase-8, −9, and −3 compared to other controls (Figure 3D). Thus, these data suggested that PAH-CuS and LPS-CuS with laser irradiation effectively induced apoptosis of CT26 cells.

Next, we evaluated anti-tumor effect of LPS-CuS with laser irradiation in CT26 tumor-bearing BABL/c mice. BALB/c mice were subcutaneously (*s.c*.) injected with 1 × 10^6^ CT26. Once CT26 tumors were well established on day 7, the mice received an intratumoral (*i.t*.) injection of PBS, 5 mg/kg PAH-CuS, and 5 mg/kg LPS-CuS. The mice were irradiated with an 808 nm laser at 1 W/cm^2^ for 5 min 2 h after the treatment. The irradiation of laser in PAH-CuS and LPS-CuS-treated tumor induced marked increase of temperature in the tumor tissue (58.5°C ± 0.9°C and 59.8°C ± 1.2°C, respectively), whereas PBS treatment under laser irradiation did not increase the temperature (Supplementary Figure 6). On day 21 of tumor injection, the tumors disappeared in the mice after treatment with PAH-CuS and LPS-CuS with laser irradiation (Figure 3E and 3F). These data suggested that PAH-CuS and LPS-CuS with laser irradiation promoted PTT-mediated anti-cancer effect against CT26 cells.

### Immune stimulatory effect of LPS-CuS

LPS is a well-known immunostimulatory molecule [24]. Since we injected LPS-CuS in tumor tissue, we first examined whether LPS can detach from PAH-CuS into a free form to reach tumor-draining lymph node (drLN). Due to the LbL-fabrication, LPS will be pH-responsively released from NPs within cancerous cells, where pH is acidic [35]. The levels of negative charge on LPS-CuS were markedly decreased at pH 5 (Supplementary Figure 7), which indicated LPS was detached from PAH-CuS.

**Figure 4:**
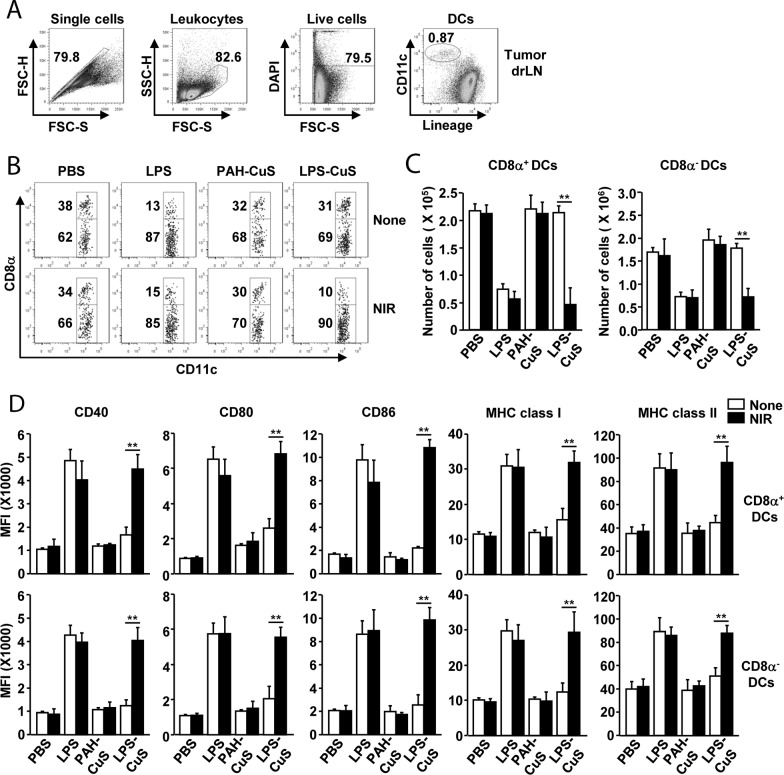
LPS-CuS with laser irradiation promoted DC activation in tumor draining lymph node (drLN) CT26 tumor-bearing mice were intratumorally injected with PBS, LPS, PAH-CuS, and LPS-CuS for 2 h, and treated with or without laser irradiation for 5 min. Tumor drLN were harvested 24 h after treatment. **(A)** Definition of DC population in tumor drLN. Lineage markers included CD3, Thy1.1, B220, Gr-1, CD49b, and TER-119. **(B)** Lineage-CD11c^+^ DCs were further divided as CD8α^+^ and CD8α^−^ DCs. **(C)** Mean number of CD8α^+^ and CD8α^−^DCs in tumor drLN. **(D)** Mean fluorescence intensity (MFI) of co-stimulatory molecules and MHC class I and II in gated CD8α^+^ and CD8α^−^ DCs in tumor drLN were analyzed using flow cytometry. All data are representative of or the average of analyses of six independent samples (i.e., three samples per experiment, two independent experiments).

Because LPS can detach from PAH-CuS and release from apoptotic cells, we next examined whether free form of LPS can induce DC stimulation in tumor-drLN and spleen. CT26 tumor-bearing BLAB/c mice were *i.t*.-injected with PBS, 0.1 mg/kg LPS, 5 mg/kg PAH-CuS, and 5 mg/kg LPS-CuS and after 2 h, laser irradiation (1 W/cm^2^) was performed for 5 min. Tumor-drLN and spleen were harvested and analyzed for the activation of DCs and its subsets 24 h after treatment. The population of DCs in the tumor-drLN and spleen were defined as lineage^−^CD11c^+^ cells in live leukocytes (Figures 4A and Supplementary Figure 8). LPS-CuS treatment with laser irradiation promoted dramatic decreases in the frequency of CD8α^+^ DCs and increases in the population of CD8α^−^ DCs. The total number of CD8α^+^ and CD8α^−^ DCs were significantly decreased by LPS-CuS treatment with laser irradiation, which decreased the levels of DC numbers almost similar to that achieved by LPS treatment alone (Figure 4B and 4C).

Next, we also examined the activation marker expression in DCs and found that the expression of co-stimulatory molecule and major histocompatibility complex (MHC) class I and II were substantially increased by LPS-CuS treatment with laser irradiation in the both CD8α^+^ and CD8α^−^ DCs in tumor drLN (Figure 4D). Moreover, consistent with DC activation in tumor drLN, the expression levels of activation molecules in the spleen DCs were also markedly up-regulated by LPS-CuS with laser irradiation (Supplementary Figure 8). The effect of DC activation by LPS-CuS with laser irradiation was dependent on toll-like receptor 4 (TLR4), as indicated LPS-CuS treatment with laser irradiation did not induce up-regulation of co-stimulatory molecules in TLR4-knock out mice (Supplementary Figure 9). Furthermore, the treatment of LPS-CuS with laser irradiation promoted up-regulation of IL-6, IL-12p40, and TNF-α mRNA levels in tumor drLN (Supplementary Figure 10). In addition, LPS-CuS with laser irradiation induced up-regulation of IFN-γ and T-bet mRNA levels, a transcription factor of Th1 cells, while Th2 and Th17 associated mRNA was not altered (Supplementary Figure 11). Thus, these data suggested that the treatment of LPS-CuS with irradiation of laser can induce the activation of DC subsets and Th1 responses in the tumor-drLN and spleen.

### Anti-metastasis effect of LPS-CuS

**Figure 5:**
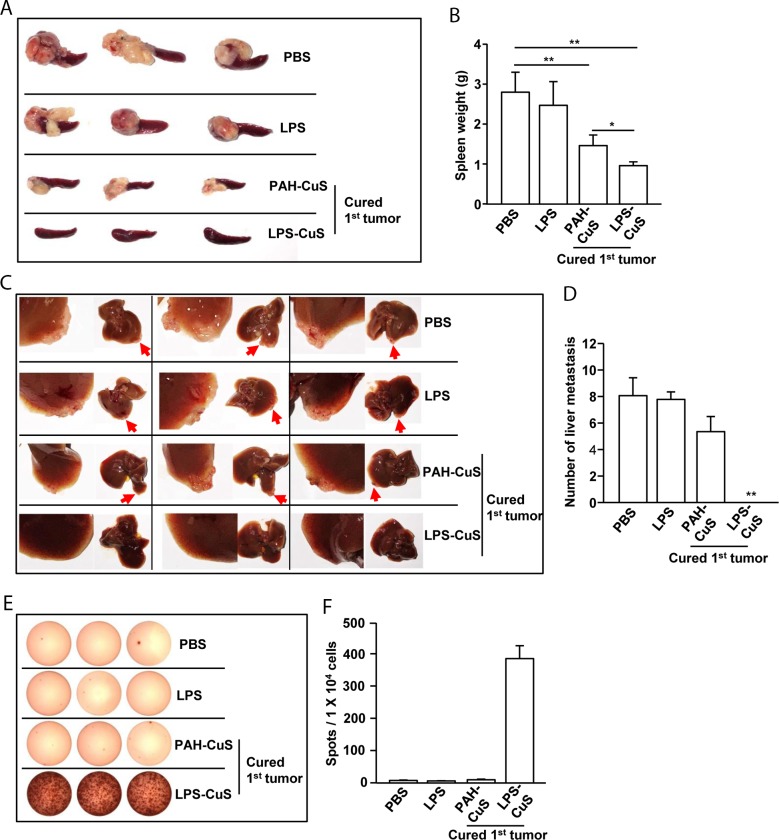
LPS-CuS treatment with laser irradiation prevented liver metastasis of CT26 cells On day 21 of primary tumor challenge, PAH-CuS and LPS-CuS treatment with laser irradiated mice were intrasplenically injected with secondary CT26 cells. PBS and LPS-treated mice were also intrasplenically injected with CT26 cells. **(A)** The size of tumor mass in the spleen on day 14 after secondary CT26 injection. **(B)** Mean weights of spleens. **(C)** Liver metastasis of CT26 tumor was measured. Red arrows indicated tumor mass in the liver. **(D)** The mean of the absolute number of CT26 metastasis in the livers. **(E)** Splenocytes were stimulated with CT26 self-antigen for 24 h. IFN-γ production were analyzed by ELISPOT analysis. **(F)** The mean number of spots was shown. Data are representative of analyses of six independent samples (i.e., three mice per experiment, two independent experiments).

Our data showing that LPS-CuS with laser irradiation induced production of apoptotic bodies and free form of LPS promoted DC activation prompted us to evaluate antigen-specific immune response-mediated anti-metastasis effect of LPS-CuS. On day 21 of primary CT26 challenge, the mice cured by PAH-CuS and LPS-CuS treatment with laser irradiation were further challenged through an intrasplenic injection with 0.5 × 10^6^ CT26 as the liver metastasis model of mice [36, 37]. We found that the mice cured of primary challenged tumor by PTT dramatically reduced secondary tumor growth in the spleen compared to PBS- and LPS-treated primary challenged CT26 in the mice 15 days after secondary tumor injection (Figure 5A). Compared to PAH-CuS-treated mice, LPS-CuS-treated mice more efficiently prevented secondary tumor growth in the spleen (Figure 5A). The weight of spleen in the LPS-CuS-treated mice was also significantly lower compared to other controls (Figure 5B). Moreover, metastasis of CT26 tumor into liver was completely inhibited in the mice that were cured of the primary CT26 challenge by LPS-CuS treatment with laser irradiation; PAH-CuS-treated cured mice and other control treatment showed liver metastasis (Figure 5C and 5D).

To determine whether LPS-CuS with laser irradiation induced anti-metastasis is mediated by antigen-specific immune response, splenocytes were incubated with the self-antigen of CT26 for 24 h and IFN-γ production was measured by ELISOP analysis. The mice cured by LPS-CuS with laser irradiation induced dramatic increases in the IFN-γ production in response to the self-antigen of CT26, whereas PAH-CuS-treated cured mice and PBS- and LPS-treated mice did not induce the production of IFN-γ (Figure 5E and 5F). Taken together, these data suggested that LPS-CuS treatment with laser irradiation promoted cancer antigen-specific immune activation and protected from the metastasis of the cancer.

## DISCUSSION

As well investigated in the previous studies, the most remarkable characteristic of CuS is the broad absorption in NIR region [38, 39]. Despite the fact that the peak absorption is shown around 980 nm, the usage of 808 nm laser instead of 980nm laser in the study was to present that CuS is broadly applicable, when compared to gold nanoparticles that specifically require certain range of light source [38, 39]. When considering the cost-effectiveness, CuS is the ideal material as its performance in treatment is not limited by the worth of the laser source, meaning that it can satisfactorily exhibit the photothermal effect under the irradiation of 808 nm laser, known as a readily accessible optical instrument. In addition, as keeping abreast of trend, the excuse for intratumoral injection of LPS-CuS without any targeting aspect, instead of intravenous injection, is not only to prevent the unexpected side effects, but also maximize the therapeutic effect through the intensified delivery of photothermal effect and anti-cancer effect [40].

It has been well studied that treatment of stimulatory molecules promotes the alteration of DC numbers in the spleen and LN [24, 25, 27, 41]. In case of LPS, it can induce full activation of DCs with the decreases in the DC numbers in the spleen and LN [24, 25]. We also found that treatment of LPS-CuS with laser irradiation promoted the decrease in DC numbers, which indicates the concentration of detached LPS from LPS-CuS may be enough for inducing DC activation in the spleen and tumor-drLN. Interestingly, LPS-CuS treatment without laser irradiation did not induce the decrease in the frequencies and numbers of DC subsets and the activation of DCs in the tumor drLN and spleen, indicating live tumor cells may not release LPS from cytoplasm.

IL-12 is one of regulatory cytokine for the development of Th1 and cytotoxic T1 (Tc1) response [42]. Moreover, LPS is also well known to promote Th1 and Tc1 activation [43, 44]. In line with previous studies that LPS induces Th1 and Tc1 responses [43, 44], LPS-CuS with laser irradiation induced up-regulation of IFN-γ and T-bet mRNA levels, a transcription factor of Th1 cells, while Th2 and Th17 associated mRNA was not altered. Thus these data suggested that detached LPS from LPS-CuS can induce Th1 immune responses.

We also found that the secondary injection of CT26 in the spleen was substantially inhibited in PAH-CuS-treated cured mice. Unlike with normal cell apoptosis, cancer cell apoptosis is able to induce immunogenic response and antitumor immunity [17]. However, the metastasis of CT26 into liver was not prevented by PAH-CuS treatment with laser irradiation. This failure of anti-metastasis effect of PAH-CuS may be due to less immune stimulatory effect, since cancer antigen cannot induce activation of APCs [10, 20, 21]. Although PAH-CuS induced effective killing of CT26 cells by laser irradiation, the apoptotic bodies may be not enough to induce antigen-specific CTL activation and antibody production because DCs and macrophages present low levels of cancer antigen and express low levels of co-stimulatory molecules to adaptive immune cells [45]. In contrast with PAH-CuS treatment, LPS-CuS treatment with laser irradiation completely prevented metastasis of CT26 cell in the liver. This prevention effect was mediated by antigen-specific immune responses. As we shown that PTT-produced apoptotic bodies and detached LPS promoted cancer antigen-specific immune activation, which consequently prevented metastasis of CT26 cells into the liver.

PTT-mediated cancer treatment received worldwide attention and grand interest from researchers in recent years [14, 46, 47]. In this study, we examined LPS-CuS-mediated PTT and immunotherapy against mouse colon cancer cells. For evaluation of immunotherapy and anti-metastasis, we chose mouse cancer cell line. As we efficiently demonstrated the LPS-CuS-mediated anti-cancer and anti-metastasis by PTT and immunotherapy, we will further examine the effect of LPS-CuS in humanized mice with human cancer cells in next study.

## MATERIALS AND METHODS

### Materials and measurement

All chemicals were purchased from Sigma Aldrich Chemical, Inc. (St. Louis, MO, USA), unless separately mentioned. Sodium Citrate (C_6_H_5_O_7_Na_3_·2H_2_O) was purchased from the DC Chemical Co. Ltd (Seoul, South Korea). Field emission transmission electron microscopy (FE-TEM) and electron diffraction (ED) pattern images were taken using a JEM-2100F transmission electron microscope (JEOL; Tokyo, Japan). Fourier transform infrared (FT-IR) spectra were recorded with a Spectrum GX (PerkinElmer Inc.; Waltham, MA, USA). Power X-ray diffraction (XRD) grapes were measured through an X'Pert-MPD System (Philips; Amsterdam, Netherlands). Dynamic light scattering (DLS) and zeta potential measurements were obtained by using an ELS-8000 (Otsuka Electronics Co. Ltd.; Osaka, Japan). UV-vis absorption spectra were recorded using an UV-Visible spectrophotometer (Beckman Coulter; Fullerton, CA, USA). A fiber-coupled continuous-wave diode laser (808 nm, 10 W) was purchased from Changchun New Industries Optoelectronics Technology Co., Ltd. (Changchun, China). Thermographic images were taken by a FLIR ONE (FLIR Systems, Wilsonville, OR, USA). Intracellular fluorescence imaging was observed by using a Leica laser scanning confocal microscope (Leica Microsystems; Wetzlar, Germany). Flow cytometry analysis data were obtained by using a BD LSR Fortessa (Becton Dickinson; Coppelle, TX, USA).

### Preparation of LPS-CuS

Citrate-stabilized CuS were prepared in accordance with the previously published report [48]. To coat PAH onto CuS [29], crude CuS solution (50 mL) was drop-wisely added to 50 mL of PAH solution (Mw = 17500, 2 mg/mL) under vigorous stirring; the mixture was stirred for 4 h. To isolate unreacted PAH from the prepared mixture, the sample was centrifuged at 19,000 rpm for 5 h; the dark green pellet of PAH-CuS was resuspended in 50 mL DI water. The LPS coating onto PAH-CuS was performed as reported by the literature, with slight modification [49]. PAH-CuS (50 mL) was added, in a drop-wise manner, to 50 mL of LPS solution (1 mg/mL) under sonication. Then, centrifugation was performed to isolate the LPS-CuS pallet, which was resuspended in a stock solution (5 mg/mL). LPS-CuS used for all experiments were from a single batch.

### Mice

BALB/c, C57BL/6 and TLR4-knock out mice were obtained from the Shanghai Public Health Clinical Center and kept under pathogen-free conditions. All experiments were carried out in agreement with the guidelines of the Institutional Animal Care and Use Committee of the Shanghai Public Health Clinical Center. The protocol was approved by the Committee on the Ethics of Animal Experiments of the Shanghai Public Health Clinical Center (Mouse Protocol Number: SYXK-2010-0098). Mice were sacrificed through CO_2_ inhalation euthanasia, and all efforts were made to minimize suffering.

### Cells

CT26 and L132 cells were provided by the American Type Culture Collection (Rockwile, MD, USA). The cells were cultured in 10% fetal calf serum (Gibco; Paisley, UK), 120 mg/L penicillin, and 200 mg/L streptomycin contained RPMI 1640 medium (Gibco; Paisley, UK) at 37°C and 5% CO_2_.

### *In vitro* photothermal treatment

CT26 cells (1 × 10^5^) were seeded into a 24-well plate for 24 h; after 2 h of PBS, PAH-CuS, and LPS-CuS treatment, the cells were irradiated with an 808-nm laser at 1 W/cm^2^ for 5 min.

### MTT assay

CT26 cells (2 × 10^4^) were seeded into a 96 well plate for 24 h. Then, 100 μL of freshly prepared MTT solution (5 mg/mL in PBS) was added to each well, then 100 μL of Dimethyl sulfoxide (DMSO, Gibco; Paisley, UK) was added to each well and incubated additional 4 h. The wells were analyzed by an ELISA reader at 620 nm (Labsystems Multiskan; Roden, Netherlands).

### Apoptosis assay

Cells were stained with annexin V-FITC and 7AAD in 100 μL of binding buffer for 15 min at room temperature (RT). The cells were analyzed by flow cytometry using a FACS Aria II (Becton Dickinson; San Diego, CA, USA) after 400 μL of binding buffer was added without washing.

### Tunel assay

CT26 cells were harvested and washed after treatment with PBS, PAH-CuS, and LPS-CuS with or without laser irradiation. The cells were attached to a slide glass using cytospin. Cells were fixed using 4% paraformaldehyde for 10 min and permeabilized with 0.1% Triton X-100 for 5 min and washed twice with PBS. Then, 100 μL terminal deoxynucleotidyl transferase-catalyzed deoxyuridine phosphate-nick end labeling (Tunel) reaction mixture (Roche, Basel, Switzerland) was then added, and the cells was incubated for 60 min at RT. PI buffer were additionally added during the last 15 min of incubation. Samples were analyzed by confocal microscopy (Leica Microsystems, Wetzlar, Germany).

### Western blot analysis

CT26 cells were treated with lysis buffer containing 1% Triton X-100, 10% glycerol, 137 mM NaCl, 1.5 mM MgCl2, 1 mM EGTA, and protease inhibitors. Proteins in the cell lysate were separated by 10% SDS–PAGE and transferred to nitrocellulose membranes. The membranes were incubated with a blocking buffer (10 mM Tris–HCl, 0.15 M NaCl, 0.1% NaN3, and 5% skim milk) for 1 h and stained with primary antibodies overnight at RT. The membranes were stained with the secondary antibodies for 2 h and signals were detected using ECL chemiluminescence following the manufacturer's instructions.

### *In vivo* photothermal treatment

Once tumors at their longest dimension reached a size of approximately 5.0 mm, mice were randomized into six treatment groups: PBS, PAH-CuS, LPS-CuS, with or without laser irradiation. Each NPs type was *i.t*. injected into the mice. An 808 nm NIR laser was applied to irradiate tumors under a power intensity of 1 W/cm^2^ for 5 min 2 h after injection. The temperature was recorded using an infrared camera FLR One Thermal imaging system (FLIR; Wilsonwille, OR, USA). Tumor volume was calculated by using the formula V ¼ 1/2 (L/S2), where L is the longest dimension and S is the shortest dimension.

### DC analysis

Tumor drLN and spleen DCs were analyzed as described previously [25, 27, 37]. Briefly, the tissues were digested by adding 2% fetal bovine serum (FBS) with collagenase after cutting into small fragments for 20 min. The cells were re-suspended in a 1.077 histopaque (Sigma-Aldrich) and upper layered in the 1.077 histopaque. The cells then centrifuged at 1700 g for 10 min. The light density fraction (< 1.077 g/cm^3^) was harvested and stained with the following FITC-conjugated monoclonal antibodies (mAbs) for lineage staining: anti-CD3 (17A2), anti-Thy1.1 (OX-7), anti-B220 (RA3-6B2), anti-Gr-1 (RB68C5), anti-CD49b (DX5), and anti-TER-119 (TER-119). The lineage-CD11c^+^ cells were defined as DCs, which were further divided into CD8α^+^ and CD8α^−^ DC subsets. The analysis was carried out using a FACS LSRfortessa (Becton Dickinson).

### Intrasplenic injection of CT26 for liver metastasis model

BALB/c mice were anesthetized using a ketamine mixture (10 μL ketamine HCl, 7.6 μL xylazine, 2.4 μL acepromazine maleate, and 10 μL H_2_O) that was injected into the peritoneal cavity. For the experiments, the CT26 cells (0.5 × 10^6^/50 μL) were inoculated in the spleens of the mice during open laparotomy.

### ELISPOT assay

Mouse IFN-γ ELISPOTs were performed according to the manufacturer's protocol (Biolegend). In short, splenocytes were harvested by density cuts and the cells were seeded at 50 × 10^3^ cells/well in an IFN-γ capture antibody pre-coated plate. Fresh 2 × 10^6^ CT26 were lysed by heat and frozen. After centrifugation, suspended antigen proteins were harvested, and 10 μg/mL antigen proteins were treated with CT26 cells at 37°C for 24 h. ELISPOT plates were counted automatically using a CTL ELISPOT reader (CTL Europe GmbH, Bonn, Germany).

### Statistical analysis

All statistical analysis results are expressed as the mean ± standard error of the mean. The statistical significance of differences between experimental groups was calculated using analysis of variance and either a Bonferroni post test or an unpaired Student's *t*-test. All *p*-values < 0.05 were considered significant.

## CONCLUSION

To develop advanced materials with nano science, a combination of research fields can help. To provide immuno-photothermal therapy for the treatment of tumor and prevention of cancer metastasis, we synthesized LPS-coated CuS. The LPS-CuS not only effectively treated CT26 tumor by PTT but also prevented metastasis of CT26 in the spleen and liver by cancer antigen-specific immune activation. Thus, LPS-CuS will be a promising candidate for the treatment of cancer and the prevention of its metastasis as an immuno-photothermal therapy material.

## SUPPLEMENTARY MATERIALS FIGURES


