# Supplementary material for: Lipopolysaccharide-coated CuS nanoparticles promoted anti-cancer and anti-metastatic effect by immuno-photothermal therapy
Source: Oncotarget. 2017 Nov 6;8(62):105584–95. doi: 10.18632/oncotarget.22331 (PMC5739661; doi:10.18632/oncotarget.22331)
Supplement: Supplementary file 1 [file oncotarget-08-105584-s001.pdf]

Lipopolysaccharide-coated CuS nanoparticles promoted anti-cancer and anti-metastatic effect by immuno-photothermal therapy

SUPPLEMENTARY MATERIALS

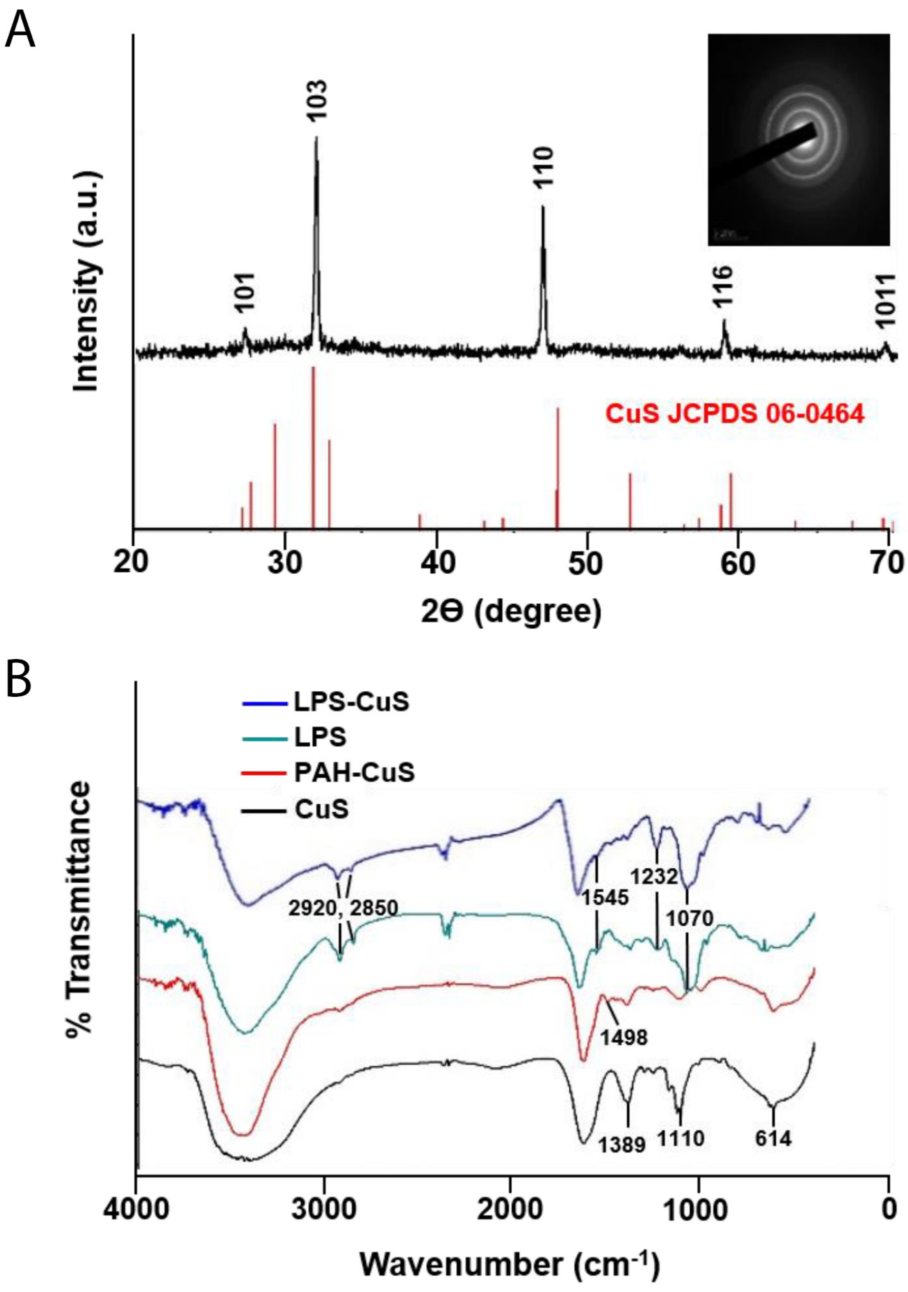

Supplementary Figure 1: Characteristics of LPS-CuS. (A) XRD pattern of CuS with the standard JCPDS card 06-0464 of CuS. **(B)** FT-IR spectra of CuS (black), PAH-CuS (red), LPS only (green), and LPS-CuS (blue).

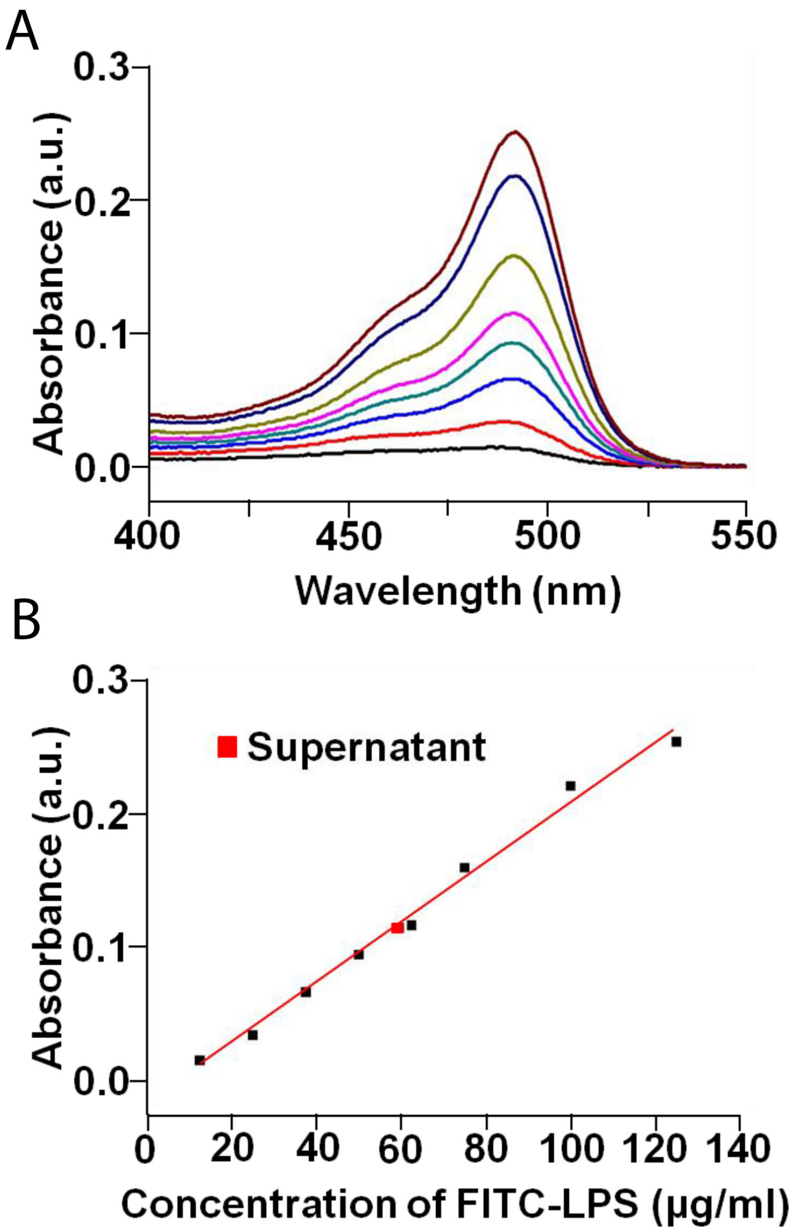

Supplementary Figure 2: Quantification of LPS coating onto NPs. (**A** and **B**) Calibration curves for the UV absorption of corresponding FITC-labeled LPS concentrations. The UV absorbance of uncoated free FITC-LPS was measured to calculate the concentration of coated LPS.

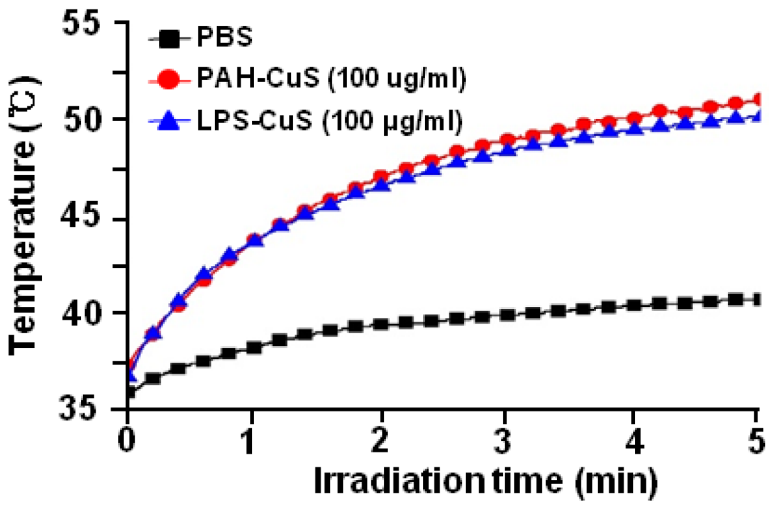

Supplementary Figure 3: Photothermal heating curves of PAH-CuS (red) and LPS-CuS (blue) with different concentration dissolved in PBS irradiated using 808 nm laser with power density of 1W/cm² for 5 min.

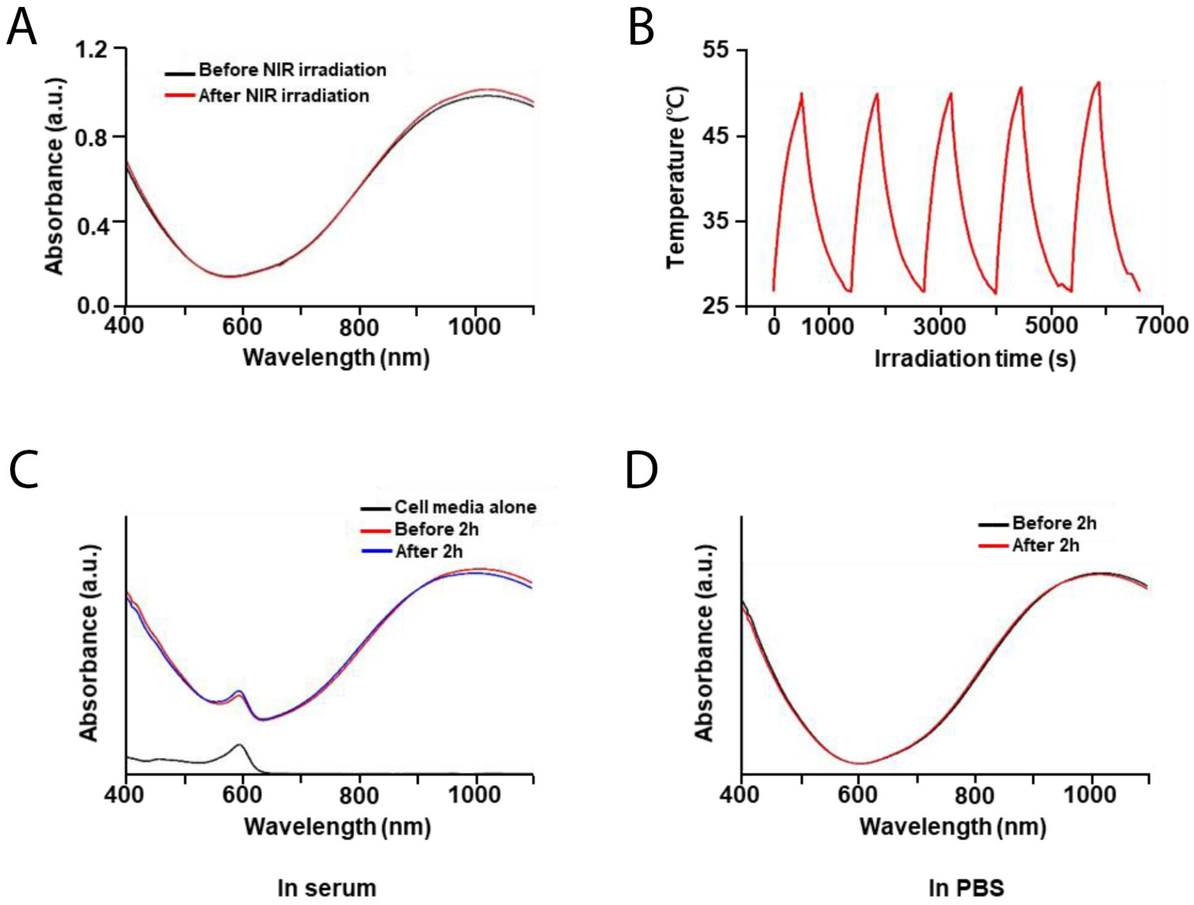

Supplementary Figure 4: Photostability test of LPS-CuS. **(A)** UV-vis absorption of LPS-CuS before (black) and after (red) NIR-irradiation. **(B)** Photothermal heating curves of LPS-CuS (50μg/mL) during the successful 5 cycles of the laser ON/OFF mode (808 nm laser, 1W/cm²). The stability of LPS-CuS in **(C)** serum contained culture medium and **(D)** PBS were measured by UV-vis absorption.

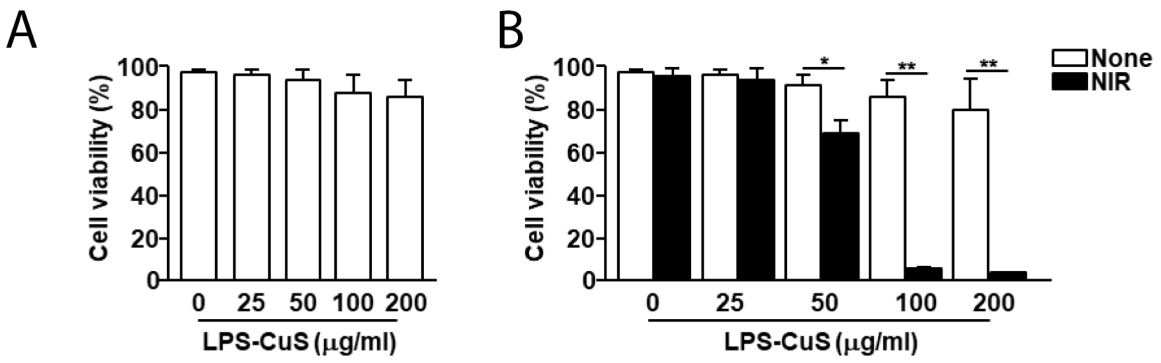

Supplementary Figure 5: Cytotoxic effect of LPS-CuS. (A) L132 cells were treated with an indicated concentration of LPS-CuS for 24 h. **(B)** CT26 cells (2 × 105) were treated with an indicated concentration of LPS-CuS, and the cells were irradiated for 5 min with an 808 nm laser. Cell viability was analyzed by MTT assay.

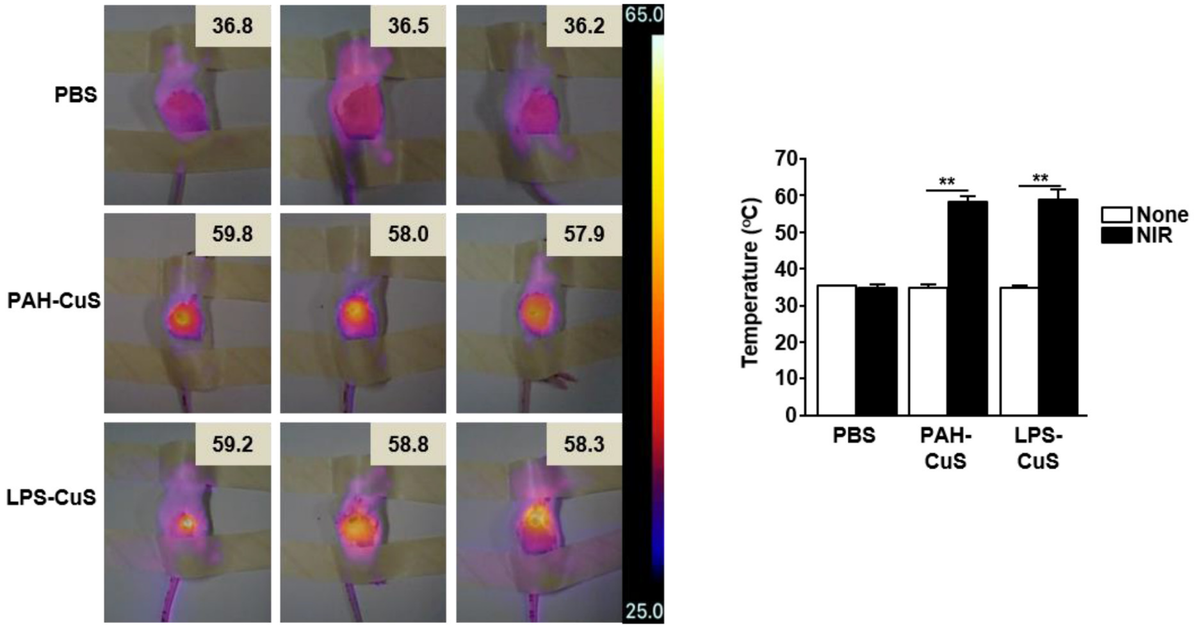

Supplementary Figure 6:Phoththermal effect of LPS-CuS *in vivo*. BABL/c mice were subcutaneously (*s.c.*) injected with 1 x 10^6 CT26. Once CT26 tumors were established on day 7, the mice received an intratumoral (*i.t.*) injection of PBS, 5 mg/kg PAH-CuS and 5 mg/kg LPS-CuS. Two hours after treatment, the mice were irradiated with 808 nm laser at 1 W/cm^2 for 5 min and the temperature increases were observe by IR camera.

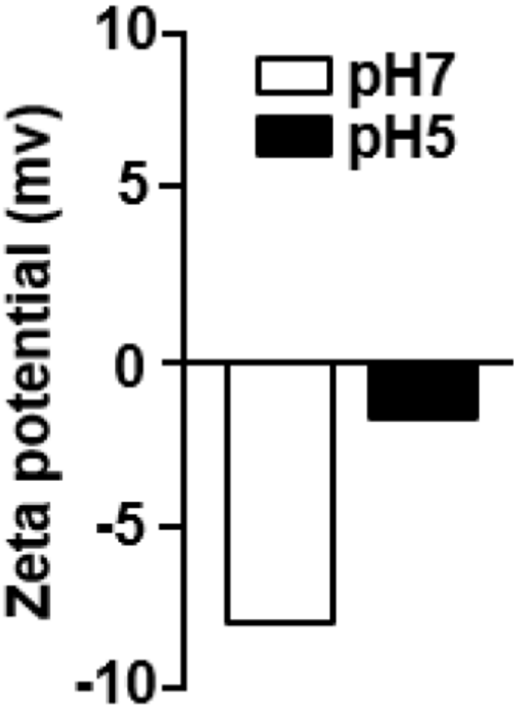

Supplementary Figure 7: Release feature of LPS from NPs. Zeta potential value of LPS-CuS changed from -8.23 (at pH 7) to -2.26 (after 2h exposure at pH 5).

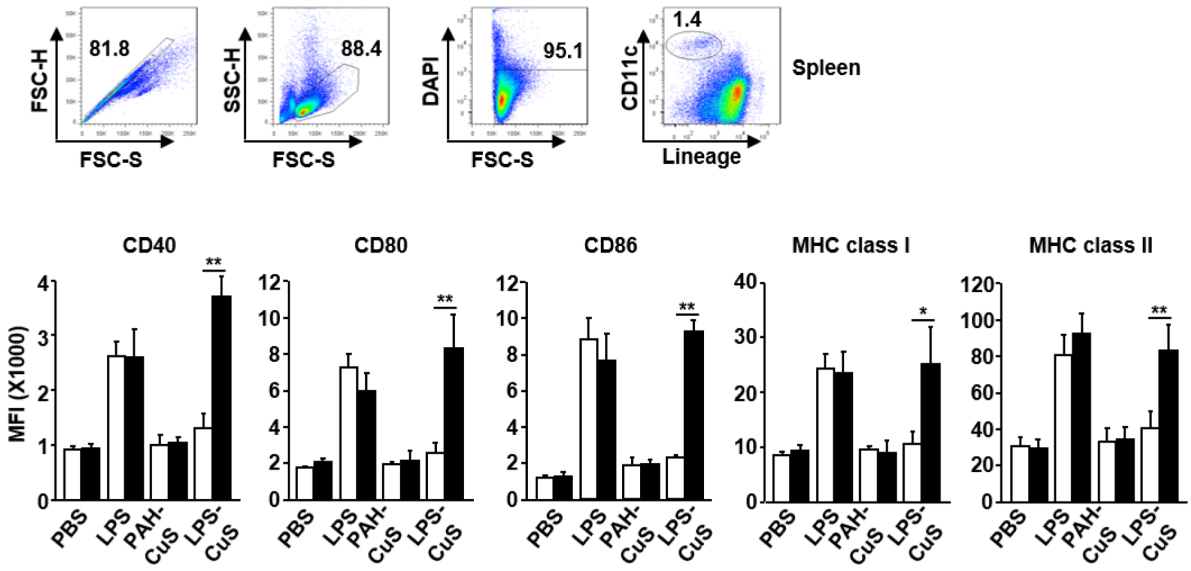

Supplementary Figure 8: LPS-CuS treatment with laser irradiation promoted activation of spleen DC. CT26 tumor-bearing BABL/c mice were intratumorally injected with PBS, PAH-CuS and LPS-CuS for 2 h and treated with or without laser irradiation. Twenty-four hours after treatment the spleen were harvested and measured activation marker on the surface. The spleen DCs were defined as a lineage-CD11c+ cells in live leukocytes (upper panel). MFI of co-stimulatory molecules and MHC class I and II expression were shown (lower panel). All data are representative of, or the average of, analyses of six independent samples (i.e., three samples per experiment, two independent experiments).

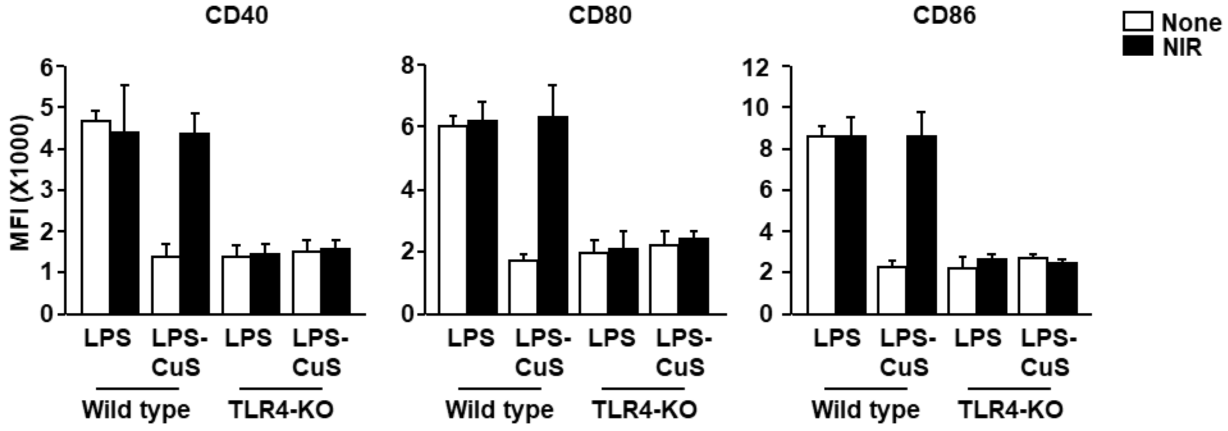

Supplementary Figure 9: TLR4 dependent DC activation by LPS-CuS. C57BL/6 mice (wild type) and TLR4-KO mice were s.c. inoculated 1 X 10^6 B16 cells. Once the tumors were established on day 10, the mice were treated with LPS and LPS-CuS for 2 h and irradiated with laser for 5 min. The DC activation in tumor drLN were analysed by flow cytometry after 24 h of treatment. All data are representative of, or the average of, analyses of six independent samples (i.e., three samples per experiment, two independent experiments).

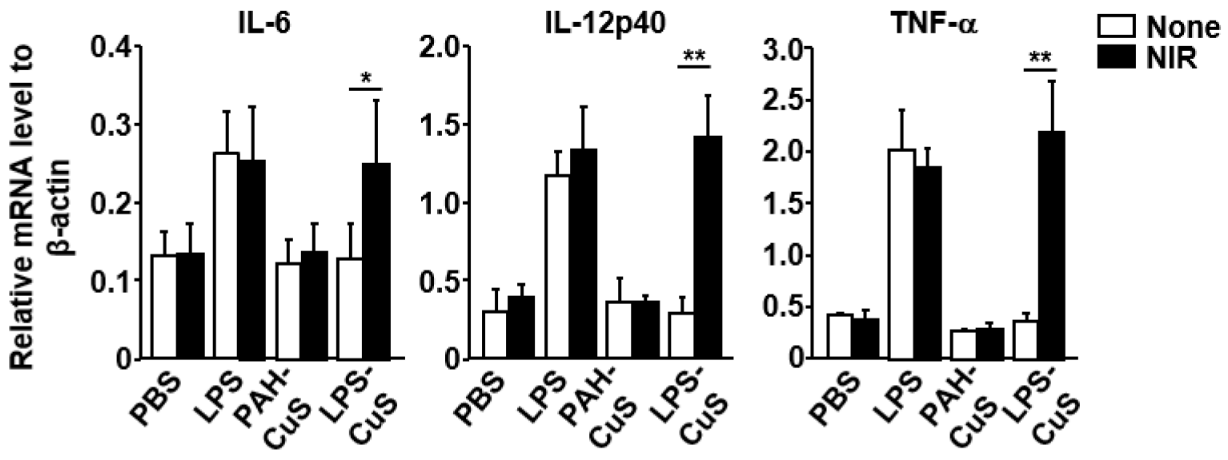

Supplementary Figure 10: LPS-CuS treatment with laser irradiation promoted up-regulation of mRNA of pro-inflammatory cytokines. The mRNA of indicated cytokines in tumor draining lymph node (drLN) was measured by Real-time PCR 24 h after treatment of PBS, LPS, PAH-CuS and LPS-CuS with or without laser irradiation. Data are the average of analyses of six independent samples.

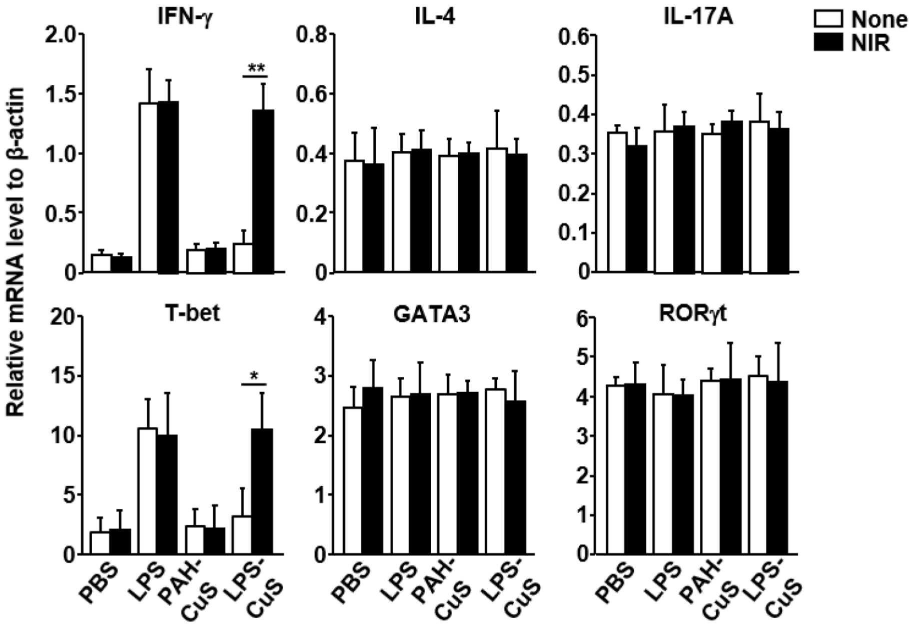

Supplementary Figure 11: LPS-CuS treatment with laser irradiation induced Th1 immune responses. The mice were treated with PBS, LPS, PAH-CuS and LPS-CuS as indicated in Figure 4. The mRNA levels in the spleen were measure 24 hour after treatment. The data are the average of analyses of six independent samples.
